# Know where to go: evidence from a controlled trial of a healthcare system information intervention among immigrants

**DOI:** 10.1186/s12889-018-5741-x

**Published:** 2018-07-11

**Authors:** Signe Smith Jervelund, Thomas Maltesen, Camilla Lawaetz Wimmelmann, Jørgen Holm Petersen, Allan Krasnik

**Affiliations:** 1 Department of Public Health, Section for Health Services Research, Danish Research Centre for Migration, Ethnicity, and Health, University of Copenhagen, Øster Farimagsgade 5A, DK-1014 Copenhagen K, Denmark; 2 Department of Public Health, Section for Biostatistics, University of Copenhagen, Øster Farimagsgade 5A, DK-1014 Copenhagen K, Denmark

**Keywords:** Intervention, Migrants, Information, Healthcare system, Healthcare utilisation, Healthcare-seeking behaviour, Innovation, Integration, Health literacy, Health education

## Abstract

**Background:**

Immigrants may face problems with accessing the Danish healthcare system due to, for example, lack of knowledge of how to navigate it, which may cause inappropriate healthcare-seeking. Danish municipalities provide a mandatory introduction and language programme for newly arrived immigrants, but no information on the healthcare system is offered. This study investigated what effects information about the Danish healthcare system may have on the hypothetical healthcare-seeking behaviour of newly arrived immigrants and their actual healthcare use.

**Methods:**

A prospective intervention study of 1572 adult immigrants attending two language schools in Copenhagen was carried out. Two intervention groups received either a course or written information on the Danish healthcare system, respectively, while the control group received neither. Survey data included three case vignettes on healthcare-seeking behaviour (flu-like symptoms, chest pain and depression) and were linked to registry data on sociodemographic characteristics and healthcare use in the year to follow. Logistic regression and binomial regression analyses were performed.

**Results:**

Appropriate hypothetical healthcare-seeking behaviour was reported by 61.8–78.8% depending on the vignette. Written information showed no effect on immigrants’ hypothetical healthcare-seeking behaviour, while the course showed a positive effect on hypothetical healthcare-seeking behaviour for flu-like symptoms (adjusted odds ratio [AOR] = 1.71, 95% confidence interval [CI] = 1.01–2.91, *p*-value = 0.0467), but not on chest pain or depression. The interventions did not affect immigrants’ actual healthcare use; all groups made lower use of health care services in the following year compared with the year where the study took place, except for the use of dental care which remained stable.

**Conclusions:**

Information on the healthcare system embedded in the language school programme has the potential to facilitate immigrants’ access to healthcare. Yet, the results underscore the need for further refinement and development of educational interventions, as well as ensuring adequate utilisation of healthcare services by other means. Multi-dimensional and multi-sectional efforts are important for integration issues within healthcare in Europe.

**Trial registration:**

Health-seeking behaviour among newly arrived immigrants in Denmark ISRCTN24905314, May 1, 2015 (Retrospectively registered).

## Background

Equity in access to healthcare is one of the fundamental values of the European healthcare systems [1]. Nevertheless, recently arrived immigrants may face several problems with accessing the healthcare system in terms of informal barriers such as language problems and lack of knowledge of how to navigate the healthcare system [2]. As a result, immigrants may show inappropriate and suboptimal healthcare-seeking behaviour and utilisation of healthcare services, which are likely to impede health among immigrants and lead to poor(er) and possibly more costly outcomes both for the individual and for society. Hitherto, in relation to the present study, we investigated how healthcare education was unfolded in the language school, and whether systematic healthcare education affected immigrants’ knowledge of the Danish healthcare system. An explorative qualitative process evaluation elucidated how the participants’ understanding of health and their risks perception overruled the normative information they learned on the healthcare education course [3]. Nevertheless, we found that healthcare education improved knowledge of the Danish healthcare system in terms of who to contact in the event of an accident but not in the event of illness and further, it positively affected correct answers for 9 out of 11 true or false questions on access to and use of the healthcare system [4]. The next step is to investigate whether improved knowledge leads to more optimal healthcare-seeking behaviour and healthcare utilisation.

Healthcare-seeking behaviour can be understood as a person’s process of engaging (or not) with a particular health service. This includes aspects such as how symptoms are perceived and acted upon and how, as well as which type of and when, healthcare services are accessed [5]. Healthcare utilisation can be seen as an interrelated result of, as well as a feedback mechanism to, healthcare-seeking behavior [6]. Inappropriate and suboptimal healthcare-seeking behaviour among immigrants has been reported previously by both healthcare professionals [7–9] and newly arrived immigrants themselves [10]. Danish healthcare professionals reported that emergency room (ER) visits by patients of Middle Eastern origin were often less appropriate in nature as compared with those by ethnic Danes [8], while immigrants themselves reported lack of knowledge of how to access the healthcare system [10]. This is in line with the results from a recent European review which showed that non-EU immigrants used ER services more frequently and were more likely to use the ER during unsocial hours and for low-acuity medical reasons as compared with native-born Europeans [11]. In Denmark, it has been demonstrated that non-Western immigrants also have higher utilisation of ER, hospital care, specialist doctors and general practitioners (GPs) after adjusting for indicators of health needs [12, 13], but lower utilisation of preventative services such as screening [14], dental care [12] and vaccinations [15–17].

The Danish healthcare system is tax-based and offers universal access and free-of-charge services to all 5.8 million inhabitants, except for certain services such as dental treatment and medicine requiring co-payment [18]. Denmark has seen a steady increase in immigration over the past thirty years, with 58% of immigrants originating from non-Western countries. As of 2018, more than 10% of the population of Denmark consists of immigrants [19] with the majority originating in Poland, Syria, Turkey, Germany, Romania, and Iraq [20]. Danish municipalities provide a free Danish language education (equivalent to 1.2 years full-time) as part of the introduction programme for all newly arrived immigrants in order to support them in obtaining the necessary Danish language skills and gaining knowledge of the culture and society in Denmark with a special focus on work, education and democracy. The aim is that immigrants are equipped to participate in and contribute to society on an equal footing with other citizens [21]. The Danish language education is a compulsory part of the introduction programme for immigrants from countries outside the EU who are granted residence permits in Denmark due to family reunification or asylum [22]. However, no introduction to the Danish healthcare system is offered or included in the introduction programme. This could otherwise be a natural part of the introduction programme to ensure that immigrants are equipped to understand and navigate the healthcare system— a sector which everybody comes into contact with (several times a year for many people) and for which access is often vital—on an equal footing with other citizens. Additionally, good mental and physical health is a prerequisite to active engagement in society and for successful integration, including the acquisition of a new language and labour market attachment [23]. Better integration may also improve health outcomes, as immigrants increasingly have the ability to seek healthcare when needed and tend to achieve life conditions that are less harmful to their health [23].

Given the high number of new immigrants in Denmark [24] and in other European countries [25] combined with their often high need for healthcare [26, 27], the observed suboptimal healthcare-seeking behavior [7, 8, 10] and healthcare use among some immigrant groups [11–13, 28] as well as barriers to access [2] calls for sustainable interventions that improve access to and appropriate use of healthcare services for immigrants, preferably at a low cost [29]. It has been shown that education is effective in increasing health knowledge and health literacy [30], which is the capacity to seek, understand and act on health information [31]. Swedish researchers have also concluded that more health and healthcare system education for immigrants would be suitable to increase their access to the healthcare system [32]. This is likewise requested by immigrants themselves [33]. Ekblad et al. found that a relatively short and culturally tailored lifestyle course strengthened self-rated heath and prerequisites for increased health literacy among newly arrived female immigrants from non-EU countries [32]. However, only few and often unsystematic initiatives have been taken, and a lack of knowledge exists on how to most successfully educate immigrants (e.g. by written information, by teaching, during cultural mediators, etc.). Since the majority of modes of delivering healthcare education have been evaluated based on the majority population [34], and since different population groups may use different channels for health information [35], we also need to test different modes of healthcare education [36] on immigrants. This is the first study, to our knowledge, which investigates systematic healthcare system education targeted at immigrants, including different modes of education, to improve general access to and appropriate use of healthcare services among immigrants.

### Objectives

With the overall aim to improve access to and use of healthcare services among immigrants, this study investigated whether an intervention providing two different kinds of systematic information (written information and a course) on the Danish healthcare system within a language school programme affected hypothetical healthcare-seeking behaviour and healthcare utilisation among immigrants, and which mode of information (written information or a course) could be proven to be most successful. The specific objectives were to compare the effect of a course on access to and use of healthcare services, the effect of an intervention only consisting of written material on the Danish healthcare system and the effect of no information (current situation) on newly arrived immigrants’ self-reported hypothetical healthcare-seeking behaviour and actual healthcare utilisation. Thereby, it is an educational intervention which seeks to modify healthcare-seeking behaviour among immigrants. Based on substantial research showing that written information alone is unlikely to lead to a measurable change in behavior [34] and that patient education can lead to reduction in ER utilization [36], the hypothesis was that only the course would affect immigrants’ hypothetical healthcare-seeking behaviour and reduce ER utilisation as compared to the control group.

## Methods

### Design and setting

A prospective controlled study design was used. Immigrants attending two language schools in Greater Copenhagen in 2012 and 2013 were assigned by school class to one of the following groups: a) Control Group (no systematic information: current situation), b) Intervention Group I: (written information on the Danish healthcare system), or c) Intervention Group II (a course on the Danish healthcare system). The intervention took place at one language school where the classes were randomly selected to receive either intervention I or II, and the other language school acted as the control. By comparing baseline and follow-up data including both survey and registry data on sociodemographic characteristics and actual healthcare use by linkage using participants’ Civil Personal Registration Number (CPR number), a unique identification number which all persons with the right of residence in Denmark hold, we investigated the effect of the intervention on immigrants’ healthcare-seeking behaviour and use of healthcare services.

### Participants

**Fig. 1:**
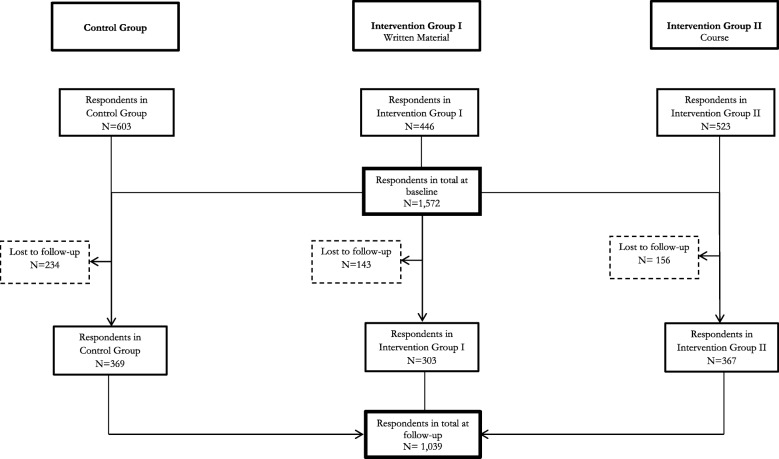
Flow diagram of the study population

Participants were immigrants defined according to the definition employed by Statistics Denmark: a person born abroad whose parents are both foreign citizens or were both born abroad [20]. Eligibility for the study was a basic understanding of Danish; thus, immigrants from the 1st Danish language module were excluded. Immigrants preparing for their final Danish language exam (module 6) were also excluded to allow them to prepare for the exam. The baseline participants comprised 1838 immigrants. Since two periods of the intervention for different groups of immigrants took place—in 2012 and 2013—some persons participated twice in the study. After omitting those who participated a second time in the study in 2013 (*n* = 217) as well as errors in CPR number (*n* = 49), the total baseline study population consisted of 1572 persons (Fig. 1). Of the 1572 participants aged 18 to 75 with a mean of 33.5 years, 63.3% were female (Table 1). 28.1% originated from Western countries (defined according to Statistics Denmark as all EU countries, Andorra, Iceland, Liechtenstein, Monaco, Norway, San Marino, Switzerland, Vatican state, Canada, USA, Australia and New Zealand [20]), 36% from Southern and Southeast Asia, 21.5% from the Middle East and North Africa, 10.7% from Sub-Saharan Africa and 3.7% other (former Soviet Union; Central and South America). Of the 1572 participants, 528 were lost to follow-up mainly due to longer school absence or leave at the time of the follow-up survey, and only an insignificant number (four persons at the intervention school in 2012) did not want to participate. The follow-up study population comprised 1039 persons and represented a response rate of 66.4% (a detailed description of follow-up study population can be found in [4]). The response rate in the Intervention Groups (69.2%) was slighter higher than in the Control Group (61.9%).

**Table 1:** Characteristics of the Study Population (baseline)

Population characteristics	Control Group *n* = 603%	*n*	Intervention Group I Written Material *n* = 446%	*n*	Intervention Group II Course *n* = 523%	*n*	Total *n* = 1572
Age (mean) (min-max)	35.1 years (18–75 years)		32.5 years (18–59 years)		33.0 years (18–75 years)		
Sex							
Female	60.3	363	67.4	300	63.8	333	996
Male	39.7	239	32.6	145	36.2	189	573
Total		602		445		552	1569
Marital status							
Married/living with partner	60.5	364	67.4	300	69.9	365	1029
Not married/divorced/widow	39.5	238	32.6	145	30.1	157	540
Total		602		445		522	1568
Geographical Origin							
Western	24.9	150	31.0	138	28.5	149	437
South and Southeast Asia	29.2	176	38.9	173	39.9	208	557
Middle East and North Africa	26.6	160	18.4	82	19.4	101	343
Sub-Saharan Africa	14.6	88	8.3	37	9.2	48	173
Other (Former Sovjet Union, Middle and South America)	4.7	28	3.4	15	3.1	16	59
Total		602		445		522	1569
Migrant Status							
Labor/study	31.8	192	34.5	154	36.1	189	535
Family reunified	48.8	294	53.8	240	51.6	270	804
Refugee	12.4	75	4.3	19	3.8	20	114
Other	1.5	9	2.7	12	2.7	14	35
NA	5.5	33	4.7	21	5.7	30	84
Total		603		446		523	1572
Citizenship							
Danish	6.8	41	-^a^	< 5^a^	-^a^	< 5^a^	51
Western	26.1	157	31.7	141	25.9	135	433
Non-Western	67.1	404	67.6	301	72.8	380	1085
Total		603		445		522	1569
Length of Stay (mean) (min-max)	6.1 years (18 d. – 26 yrs.)		3.5 years (29 d. – 23 yrs.)		3.9 years (54 d. – 26 yrs.)		
< 1 year	15.6	94	19.3	86	18.5	97	277
(1–3)	33.8	204	42.6	190	44.9	235	629
(3–5)	13.6	82	22.2	99	17.8	93	274
5+	37.0	223	15.9	71	18.7	98	392
Total		603		446		523	1572
Educational Level							
None/primary	20.2	83	6.7	30	7.7	40	192
Secondary	55.6	208	68.6	306	64.2	336	977
Tertiary	22.9	81	24.7	110	27.3	146	394
NA	-^a^	< 5^a^	-^a^	< 5^a^	-^a^	< 5^a^	9
Total		603		446		523	1572
Employment Status							
Employed	42.8	253	55.5	243	56.6	289	785
Unemployed	38.4	227	19.2	84	18.4	94	405
Student	8.3	49	13.7	60	13.1	67	176
Other	10.2	60	11.6	51	11.9	61	172
Total		589		438		511	1538
Household Income (DDK)							
< 100.000	67.5	407	55.8	249	55.6	291	947
100.000–200.000	27.4	165	28.5	127	30.6	160	452
> 200.000	5.1	31	15.7	78	13.8	72	173
Total		603		446		532	1572
Children							
0 children	48.0	289	48.5	216	51.9	271	776
1 child	17.4	105	22.9	102	21.7	113	320
2+ children	34.6	208	28.5	127	26.4	138	473
Total		602		445		522	1569
Origin of Spouse							
Denmark	-^a^	< 5^a^	-^a^	< 5^a^	-^a^	< 5^a^	6
Western	19.4	117	26.0	116	23.5	123	356
Non-Western	77.5	470	71.3	318	74.4	389	1177
No spouse	1.9	12	2.5	11	1.9	10	33
Total		603		446		523	1572

^a^Micro data/not allowed

### Intervention

Professional Danish language teachers and the project leader (SSJ) developed an information letter, questionnaire and course material in Danish (available at www.sulim.ku.dk/research/wp6/). Subsequently, the project leader pilot tested the information letter and the questionnaire through focus group discussions with five to six representatives of immigrants at different levels of Danish language proficiency, with two teachers and a study coordinator present. The pilot test did not lead to any essential revisions, except for the addition of one answer category (pray to God) to the items on healthcare-seeking behaviour in the questionnaire. The information letter included the study objectives, data collection process and anonymity procedures, and specified that the participant was free to decide whether or not to be involved in the study and could withdraw at any point. Furthermore, it highlighted that participation had no consequences for his/her legal status in Denmark, education, healthcare, social security or status/role at the language school. The questionnaire consisted of 14 overall items on type of residence permit, self-assessed and fact-based knowledge of the Danish healthcare system, case vignettes on healthcare-seeking behaviour, satisfaction with the Danish healthcare system and the participants’ perception/culture, including use of healthcare in home country. The results for knowledge and satisfaction have been reported elsewhere [4].

We informed all teachers at both language schools about the study at a plenary session in 2012, and again in 2013. The teachers at the intervention school were furthermore informed in smaller group meetings, where the questionnaire and data collection procedures were explained in more depth. The teachers who were to educate Intervention Group II on the Danish healthcare system were given training sessions and supervision to standardise the sessions before the intervention took place.

We used two intervention methods: Intervention Group I received only written information on the Danish healthcare system, while Intervention Group II received a course on the Danish healthcare system as well as the written information. The written information took the form of a 15-page booklet with pictures (a total of 8 pages of written text) at a low reading level. The text was simplified and rewritten from an original booklet issued to immigrants by the Danish National Board of Health and Danish Cancer Society, as language school teachers assessed the Danish level of the booklet too high for immigrants with limited Danish language skills. The booklet included explanations of healthcare terminology, the organisation of the Danish healthcare system, types of providers, how and when to access different providers, the principle of confidentiality for healthcare professionals, how to access interpreters, access to prescriptions and medicines, preventive healthcare services and the healthcare system culture in broad terms, including the doctor-patient relationship and mutual expectations. Using a backwards translation procedure, it was translated from Danish by professional interpreters into the eight most spoken languages at the language schools: Arabic, English, Mandarin, Thai, Urdu, Tagalog, Polish and Turkish. The immigrants chose booklets in their preferred language, which were handed out in class by their teachers.

Intervention Group II received a 12-hour course as part of their Danish language programme, taught by their usual professional language teachers. The course included the same topics as the booklet and made use of exercises as well as empirical examples that encouraged discussion. As part of the course, a GP taught one session to inform participants about the GP’s role and function, and to discuss the expectations and roles of patients and doctors as well as views on prescription medicine. Intervention Group II also received the written information in addition to the course.

To inform them about the study, all participants received the information letter at school two weeks prior to baseline data collection. The information letter was again presented to the participants at baseline data collection using the questionnaire (weeks 4–5 in January), followed by the intervention efforts (weeks 6–7 in February) and the follow-up data collection using the same questionnaire (weeks 12–22 in April/May).

During a class, the questionnaires were completed by the participants. This took approximately 45 min. Teacher assistance was offered when a participant did not understand a question. For the participants at the lowest educational level, of whom many were illiterate, the questionnaire was read aloud to all and they completed the questionnaire themselves with guidance. The teachers and the study coordinator kept track of all potential participants, and if a person was not present on the day of data collection, he/she was asked to fill in the questionnaire at a help desk during class in the following two to three weeks in order to obtain a sufficient participation rate. At the time of follow-up data collection, 18 persons had shifted their intervention groups due to changing their classes; these persons were categorised according to their baseline intervention group.

The questionnaire information was subsequently linked to registry information on sociodemographic characteristics, length of stay in Denmark and use of healthcare services. Type of residence permit (labour migrant; family reunified; refugee) was obtained from the questionnaire, since self-assessment of this information was considered more reliable than registry information and was thought to reflect the participants’ own perceptions of their reasons for immigration. Information on education level was obtained from the language school, as this information is lacking in the registries for a large number of immigrants. We also believed that information from the language school would be more accurate, as the schools have comprehensive dialogues with all immigrants and test them before dividing them into classes and levels. At the language schools, educational level is based on teaching levels: 1) ‘none/primary’: illiterates and those with fewer than 7 years of primary school education, 2) ‘secondary’: those with at least a primary school education, but without a complete high school education and 3) ‘tertiary’: those with a minimum of a complete high school education.

### Main outcome measures

To assess healthcare-seeking behaviour, three case vignettes with descriptions of persons with different healthcare needs at different times of the day were used: i) flu-like symptoms; ii) chest pain; iii) major depression. The case with flu-like symptoms was described as the following: “*Sofia is 35 years old. She lives alone and is almost never ill, but she is stressed. Sofia is sleeping poorly at night. She wakes up on a Saturday morning and has a sore throat. She also coughs. Sofia takes her temperature and has 39.5 degrees in fever.*” The case with chest pain was described as the following: “*Ali is 55 years old. He is almost never ill, but he is too fat and he does not exercise. Monday at 1 p.m., Ali carries a box of oranges up the stairs. Suddenly Ali feels pain in his chest close to his heart. It hurts very much, and Ali does not feel well at all. After 5 minutes, he feels better, and in the end, he is fine. Sometimes, Ali has the same pain when he has eaten a lot.*” The case with major depression was described as the following: “*Kim is 28 years old. He is very sad, he cries sometimes and is in a bad mood. Kim has no desire to anything and he is always very tired. He does not sleep well at night. He cannot concentrate or remember well. One Friday evening, Kim thinks he wants to die*.” The participants were asked to report on what they would do in the three cases by only choosing one of the following answer categories: a) talk to family and friends; b) go to pharmacy; c) call 112 (the Emergency Operations Centre); d) consult an out-of-hours-doctor; e) go to ER; f) contact GP; g) pray to God; h) nothing. To assess the “appropriateness” of service use, we applied an understanding of accessing healthcare services according to health need by the principle of using the lowest effective care level from the lens of a Western medicine oriented healthcare system. In the case of *flu-like symptoms with relative high fever,* where consulting a doctor may be appropriate but in most cases does not require emergency healthcare, the following actions were categorised as appropriate healthcare-seeking behaviour: consult out-of-hours-doctor and go to GP. Inappropriate healthcare-seeking behaviour was considered as: talk to family and friends, go to pharmacy, call 112, go to ER, pray to God and no actions. In the case of *chest pain,* where fast healthcare might be life-saving, the following actions were considered as appropriate healthcare-seeking behaviour: call 112, go to ER and go to GP. Inappropriate healthcare-seeking behaviour was considered as: talk to family and friends, go to pharmacy, consult out-of-hours-doctor, pray to God and no actions. Consulting an out-of-hours doctor was considered inappropriate as the case took place during a weekday at 1 pm where out-of-hours doctors are not available. In the case of *major depression* that requires healthcare or help to seek healthcare, the following actions were considered as appropriate healthcare-seeking behaviour: talk to family and friends, consult out-of-hours-doctor, go to ER and go to GP. Inappropriate healthcare-seeking behaviour was considered as: go to pharmacy, call 112, pray to God and no actions. We also carried out sensitivity analyses for all three cases, where we added “pray to God” to the appropriate answer category as well as “talk to friends and family”, “go to pharmacy”, and “do nothing” for the case of flu-like symptoms.

Utilisation of healthcare services was assessed by calculating the contacts to healthcare services a year prior to and the year after the intervention took place. The healthcare services contacts we assessed were as follows: ER (number of contacts), GP (number of contacts either telephone, email or in-person consultation), outpatient treatment at hospital (contact versus no contact), inpatient treatment at hospital (contact versus no contact), specialist doctor in private practice which needs referral from GP (contact versus no contact) and dentist (contact versus no contact).

### Statistical methods

An ‘intention-to-treat’ approach was used. To compare the baseline versus follow-up changes in healthcare-seeking behaviour using the follow-up study population (*n* = 1039), the answer categories with flu-like symptoms, chest pain and major depression were dichotomised into “right” (appropriate) or “wrong” (inappropriate) both at baseline and follow-up. McNemar’s test for binary-outcomes was used to test changes in healthcare-seeking behaviour between baseline and follow-up in the three groups (Control, Intervention Group I and Intervention Group II) separately. Conditional logistics regression was then used to compare the groups (Control, Intervention I and Intervention II). This analysis utilises a design in which each individual acts as his/her own control; thus, we were able to adjust the model for different covariates: age, sex, marital status, type of migration, length of stay in Denmark, citizenship, country of birth, education, employment status, household income, number of children and origin of spouse. The results of the conditional logistics regression are interpreted as follows: the change in the control group is an odds ratio that is the odds of right response at follow-up divided by the odds of right response at baseline. This is the placebo effect or the effect of time or experience, etc. The change in the intervention group is similarly given by an odds ratio. The ratio between the two odds ratios is an estimate of the true intervention effect. The effect can also be interpreted as an individual causal effect: the improvement that a hypothetical individual would experience if he or she were to be investigated in both a placebo setting and an intervention setting.

To assess the intervention’s effects on healthcare utilisation, we used the baseline study population (*n* = 1572) as the outcome measures stemmed from registry data only. For the number of emergency room contacts and the number of visits to the GP, we used binomial regression at individual level since both measurements’ time points are poisson distributed and second measurement conditioned on the sum of the two measurements is binomial distributed. The estimate is a rate ratio (RR) because we obtain the ratio between the average at the second time point with the first at individual level. For the dichotomised outcomes for specialist doctors, dentists, inpatient and outpatient hospitalisation, we used conditional logistic regression. The regressions were performed in the same way as for the analyses on healthcare-seeking behaviour when adjusting for covariates. The statistical software SAS (version, 9.4) was used for the analyses.

### Data protection agency approval and ethical considerations

The Danish Data Protection Agency granted permission for the study. According to the Danish Act on a Biomedical Ethics Committee System and the Processing of Biomedical Research Projects, this study was not notifiable to the Danish Research Ethics Committee System, as it did not include biological material. All potential participants received written information about the study (the information letter) underscoring study objectives, anonymity procedures, participants’ rights to withdraw and that (non-)participation had no consequences for the individual. The intervention groups received further oral information from their teachers about the study where it was possible to ask questions. According to national regulations, filling in the questionnaire was considered an informed consent. A person-encrypted database comprising both questionnaire and registry data to be used for the analyses was created by Statistics Denmark.

## Results

The characteristics of the baseline study population by intervention group are depicted in Table 1. The mean age of the participants was 32.5–35.1 years, depending on the group, and the majority were women (60.3–67.4%) and married/living with a partner (60.5–69.9.1%). The most common regions of origin were South and Southeast Asia (29.2–39.9%), Western countries (24.9–31.0%) and the Middle East and North Africa (18.4–26.6%). The main reasons for Danish residence permits were family reunification (48.8–53.8%) and labour/study (31.8–36.1%). The participants’ mean length of stay in Denmark was 3.5–6.1 years. In regards to socioeconomic factors, the majority had a secondary level education (55.6–68.6%), were employed (42.8–56.6%) and had an equivalent household income below 100,000 DKK (55.6–67.5%) which is a low household income in Denmark. The participants’ education level, employment status, household income and migrant status differed somewhat by intervention group with lower level of education, more unemployed, lower household income and more refugees in the Control Group compared with the two intervention groups.

### Effects on hypothetical healthcare-seeking behaviour

#### Flu-like symptoms

In the case of flu-like symptoms, appropriate hypothetical healthcare-seeking behaviour was reported at baseline by 61.8% (Table 2). The most common replies on what the participants would do were: call out-of-hours-doctor (32.0%), go to GP (29.8%), go to pharmacy (9.9%), go to ER (8.6%) and call 112 (7.1%). There were no overall significant differences in the replies between the three groups, yet, more individuals in the Control Group reported that they would call an out-of-hours doctor (34.4%) compared with the intervention groups (30.5–30.6%). At follow-up, the intervention in the form of written information did not affect immigrants’ hypothetical healthcare-seeking behaviour, nor did it when adjusting for covariates. Yet, the course was found to have a borderline effect on the immigrants’ healthcare-seeking behaviour in unadjusted analysis (odds ratio [OR] = 1.67, 95% confidence interval [CI] = 0.99–2.83, Table 3). When adjusting for region of origin (OR = 1.71, 95% CI = 1.01–2.91), migrant status (OR = 1.74, 95% CI = 1.01–2.97) or educational level (OR = 1.74, 95% CI = 1.00–3.00), a positive effect of the course on healthcare-seeking behaviour was observed in Intervention Group II (Table 3). In the sensitivity analyses, we found a positive effect of the course on healthcare-seeking behaviour after adjusting for educational level (OR = 2.22, 95% CI = 1.04–4.74).

**Table 2:** Hypothetical healthcare-seeking behaviour at baseline and at follow-up for the three case vignettes (*N* = 1039)

	Control Group		Intervention Group I (Written material)		Intervention Group II (Course)	
	Baseline N	Follow-up N	Baseline N	Follow-up N	Baseline N	Follow-up N
Flu-like symptoms						
Talk to family and friends	18	23	20	25	19	21
Go to pharmacy	31	25	38	19	30	22
Call 112	22	22	15	16	30	13
Consult an out-of-hours-doctor	124	122	77	92	104	112
Go to ER	20	19	28	18	29	13
Contact GP	99	97	82	88	102	122
Pray to God	< 5	10	< 5	7	9	8
Nothing	5	8	9	13	7	11
Other^a^	16	5	11	5	5	8
Chest pain						
Talk to family and friends	37	35	24	31	32	37
Go to pharmacy	11	9	12	8	6	< 5
Call 112	63	61	43	36	70	56
Consult an out-of-hours-doctor	33	34	17	24	36	36
Go to ER	21	15	30	12	32	16
Contact GP	150	143	134	139	139	164
Pray to God	8	10	6	< 5	7	5
Nothing	5	12	< 5	8	8	5
Other^a^	5	5	6	< 5	< 5	< 5
Major depression						
Talk to family and friends	114	89	114	93	114	105
Go to pharmacy	7	7	< 5	10	5	< 5
Call 112	25	29	20	27	20	17
Consult an out-of-hours-doctor	31	38	25	28	30	40
Go to ER	9	10	9	8	20	9
Contact GP	102	104	75	76	114	117
Pray to God	21	23	12	14	18	14
Nothing	11	11	15	11	14	12
Other^a^	10	8	13	9	7	8

^a^Self-exclaimed healthcare-seeking behaviour (other than the pre-defined categories)

**Table 3:** Logistic regression analysis of the association between type of intervention and healthcare-seeking behavior in three case vignettes (N = 1039)

	OR^a^	(95% CI)	*P*-value
Flu-like symptoms			
Control Group	1.00		
Intervention Group I (Written Material)	1.51	(0.86–2.65)	0.148
Intervention Group II (Course)	1.67	(0.99–2.83)	0.055
Chest pain (potential AMI)			
Control Group	1.00		
Intervention Group I (Written Material)	0.80	(0.43–1.48)	0.473
Intervention Group II (Course)	1.39	(0.80–2.41)	0.238
Major depression			
Control Group	1.00		
Intervention Group I (Written Material)	1.00	(0.53–1.89)	0.990
Intervention Group II (Course)	1.47	(0.78–2.79)	0.236
	AOR^a^	(95% CI)	*P*-value
Flu-like symptoms			
Control Group	1.00		
Intervention Group I (Written Material)^b^	1.54	(0.88–2.72)	0.133
Intervention Group II (Course)^b^	1.71	(1.01–2.91)	0.047
Control Group	1.00		
Intervention Group I (Written Material)^c^	1.63	(0.92–2.90)	0.094
Intervention Group II (Course)^c^	1.74	(1.01–2.97)	0.044
Control Group	1.00		
Intervention Group I (Written Material)^d^	1.59	(0.87–2.84)	0.120
Intervention Group II (Course)^d^	1.74	(1.00–3.00)	0.048

^a^The change in the control group is an odds ratio that is the odds of right response at follow-up divided by the odds of right response at baseline. The change in the intervention groups is similarly given by an odds ratio. The ratio between the two odds ratios is an estimate of the true intervention effect

^b^Adjusted for region of origin

^c^Adjusted for migrant status

^d^Adjusted for level of education

Table 4 displays an example using data for Flu-like Symptoms, in which the odds ratio for the Control Group is 54/54 = 1.00 and, 77/46 = 1.67 for Intervention Group II. The progress of those in Intervention Group II is compared to that of those in the Control Group through the expression 1.67/1.00 = 1.67, which is also the estimate from the conditional logistics regression in Table 3 for Intervention Group II.

**Table 4:** Assessing Healthcare-seeking Behavior for Flu-like Symptoms: Participant’s Reports of “Right” (Appropriate) or “Wrong” (Inappropriate) Healthcare-seeking Behavior at Baseline and Follow-up

Control Group					Intervention Group I Written Material					Intervention Group II Course				
		Follow-up					Follow up					Follow-up		
Frequency		Right	Wrong	Total	Frequency		Right	Wrong	Total	Frequency		Right	Wrong	Total
Baseline	Right	152	54	206	Baseline	Right	115	37	152	Baseline	Right	146	46	192
	Wrong	54	52	106		Wrong	56	57	113		Wrong	77	41	118
	Total	206	106	312		Total	171	94	265		Total	223	87	310
OR = 1.00 (95% CI = 0.69–1.46)			*P* = 1.000		OR = 1.51 (95% CI = 0.86–2.65)			*P* = 0.148		OR = 1.67 (95% CI = 0.99–2.83)			*P* = 0.055	

#### Chest-pain

Regarding healthcare-seeking behaviour for chest-pain at baseline, appropriate hypothetical healthcare-seeking behaviour was reported by 73.6% (Table 2). The most common replies on what they would do in the case of chest pain were: go to GP (45.7%), call 112 (18.8%), talk to family or friends (9.4%), call out-of-hours doctor (9.1%) and go to ER (9.1%). There were no significant differences in the replies between the three groups. At follow-up, the interventions were not found to have any effect on the immigrants’ hypothetical healthcare-seeking behaviour (Table 3). Adjusting for the covariates did not alter the conclusions (Table 3). In the sensitivity analyses, the same pattern with no effect of the interventions was observed, also after adjusting for covariates (results not shown, but available on request).

#### Major depression

In the case with major depression, appropriate hypothetical healthcare-seeking behaviour was reported by 78.8% (Table 3). The most common replies on what they would do at baseline were: talk to family or friends (36.6%), go to GP (28.9%), call out-of-hours doctor (9.3%) and call 112 (7.5%). The three groups differed in their statistically significant replies (*p* = 0.0185), with more persons in Intervention Group II replying to go to the GP (33.3%) compared with Intervention Group I (26.6%) and the Control Group (26.7%). At follow-up, the interventions were not found to have any effect on the healthcare-seeking behaviour of the participants (Table 3). Adjusting for the covariates did not alter the conclusions (Table 3). In the sensitivity analyses, the conclusions remained similar, also after adjusting for covariates (results not shown, but available on request).

### Effect on actual healthcare-utilisation

#### GP and ER

For use of GP and ER, a decreased use was detected from baseline to follow-up in all three groups (Table 5). For example, in the Control Group, a total number of 3662 contacts were made to the GP among the 500 individuals consulting their GP in the year before the intervention, and a total number of 2376 contacts were made with the GP among the 313 individuals consulting their GP in the year following the intervention (*p* < 0.0001). In both Intervention Groups a smaller decrease in the use of GPs was observed compared with the Control Group, but this was only statistically significant in Intervention Group I. In both Intervention Groups, a smaller decrease in the use of GPs and ERs was observed compared with the Control Group, albeit not statistically significantly. Adjusting for the covariates did not alter the conclusions.

**Table 5:** Use of Healthcare presented by total Number of Contacts to General Practitioner and Emergency Room by Intervention Group (*N* = 1572)

Type of Healthcare Use	Control Group							Intervention Group I Written Material							Intervention Group II Course						
	Baseline	N	Follow-up	N	RR	95% CI	P-value	Baseline	N	Follow-up	N	RR	95% CI	P-value	Baseline	N	Follow-up	N	RR	95% CI	P-value
General practitioner	3662	500	2376	313	0.65	0.62–0.68	< 0.0001	2343	359	1592	241	1.05	0.96–1.14	0.270	2292	398	1890	295	1.27	1.17–1.38	< 0.0001
Emergency Room	100	80	60	52	0.60	0.44–0.83	0.002	75	55	42	37	0.93	0.57–1.53	0.785	95	75	61	44	1.07	0.68–1.68	0.770

#### Specialist doctor, hospital, outpatient hospital visit and dentist

For specialist Doctor, Hospital, Outpatient Hospital Visit and Dentist, the majority of participants did not use any of the healthcare services, either before or after the intervention took place (Table 6). We observed that all groups made lower use of health care services in the following year compared with the year where the study took place, except for the use of dental care which remained stable. At follow-up, the interventions were not found to have any effect on the immigrants’ actual use of these services. Adjusting for the covariates did not alter the conclusions.

**Table 6:** Use of Healthcare presented by Use or No Use to Specialist Doctor, Hospital, Outpatient Hospital Visit and Dentist by Intervention Group (N = 1572)

Control Group					Intervention Group I Written Material					Intervention Group II Course				
		Follow-up					Follow up					Follow-up		
Frequency		Use	No use	Total	Frequency		Use	No use	Total	Frequency		Use	No use	Total
Specialist Doctor														
Baseline	Use	39	67	106	Baseline	Use	14	43	57	Baseline	Use	18	46	64
	No use	42	455	497		No use	29	360	389		No use	35	424	459
	Total	81	522	603		Total	43	403	446		Total	53	470	523
OR = 0.63 (95% CI = 0.43–0.92) *P* = 0.018					OR = 1.08 (95% CI = 0.59–1.98) *P* = 0.814					OR = 1.21 (95% CI = 0.68–2.18) *P* = 0.516				
Hospital														
Baseline	Use	6	43	49	Baseline	Use	6	34	40	Baseline	Use	7	46	53
	No use	24	530	554		No use	14	392	406		No use	38	432	470
	Total	30	573	603		Total	20	426	446		Total	45	478	523
OR = 0.56 (95% CI = 0.34–0.92) *P* = 0.022					OR = 0.74 (95% CI = 0.33–1.64) *P* = 0.455					OR = 1.48 (95% CI = 0.77–2.86) *P* = 0.243				
Outpatient Hospital Visit														
Baseline	Use	36	87	123	Baseline	Use	27	76	103	Baseline	Use	37	71	108
	No use	50	430	480		No use	36	307	343		No use	55	360	415
	Total	86	517	603		Total	63	383	446		Total	92	431	523
OR = 0.57 (95% CI = 0.41–0.84) *P* = 0.002					OR = 0.82 (95% CI = 0.49–1.40) *P* = 0.473					OR = 1.35 (95% CI = 0.82–2.21) *P* = 0.237				
Dentist														
Baseline	Use	22	36	58	Baseline	Use	15	26	41	Baseline	Use	13	33	46
	No use	34	511	545		No use	26	379	405		No use	31	446	477
	Total	56	547	603		Total	41	405	446		Total	44	479	523
OR = 0.94 (95% CI = 0.59;1.51) *P* = 0.811					OR = 1.06 (95% CI = 0.52–2.17) *P* = 0.876					OR = 1.00 (95% CI = 0.50–1.96) *P* = 0.988				

## Discussion

### Key findings

This study showed that a healthcare education course had positive effects on hypothetical healthcare-seeking behaviour for flu-like symptoms but not for chest pain or major depression. Written information had no effect on participants’ hypothetical healthcare-seeking behaviour. Healthcare education or written information had no measurable effect on participants’ actual healthcare use; both intervention groups and the control group made a somewhat similar decreased use of GPs, ERs, specialist doctors, hospital and outpatient hospital visits in the year after the intervention, except for use of dental care which remained stable across the groups. Findings from this study indicate that increased knowledge of access to the healthcare system [4] has only limited or no effect on healthcare-seeking behaviour, and actual healthcare use was not shown to be affected during the following year. A key message from this study is, therefore, that we have shown that it is possible to make interventions that modify some kinds of hypothetical healthcare-seeking behavior. Other measures than healthcare educational activities alone are needed in order to ensure equity in access to healthcare services for all.

### Methodological and conceptual issues

Strenghts of the study included that we used a prospective, controlled intervention design and a control group in a real life setting, which made a wider scale/national implementation more feasible. Professional language schoolteachers who possess a great experience, insight and understanding of the target group developed the intervention material and took great effort in ensuring the immigrants’ understanding of the material. The material was further tested for reliability and validity among the target group. Professional and familiar teachers taught the educational course in a culturally sensitive manner, implying being respectful and acknowledging cultural differences and similarities without assigning them a (positive or negative) value, through dialogue and discussion. The teachers adapted the language and content of the information to the target group based on the language skills and educational levels of the participants and adapted the information to suit their interests and needs in their everyday lives. Additionally, we obtained a solid response rate among persons answering both baseline and follow-up surveys which was considerably higher than that of national baseline surveys among the general Danish population [37]. Finally, the high validity of Danish registry data on healthcare use [38, 39] as our outcome data is considered a strength together with the linkage of survey and registry data.

Limitations of this study were that the intervention was not delivered within a standardised prototype which we were able to control. Findings of the explorative qualitative process evaluation of this study elucidated how the participants’ sharing of experiences, opinions and interests modified the content in the educational course from the planned content [3]. This contributes to explaining the weak effect of the intervention. As with complex interventions, we cannot be certain that any detected difference between the groups was caused by the intervention alone [40]. Nevertheless, by using a design and statistical approach where the individual acts as his or her own control, this methodological concern is largely reduced. Neither the educational course nor the written information were designed to target an effect of the case vignettes in the questionnaire. Additionally, the case vignettes were formulated in a way that the urgency of healthcare actions was open to interpretation; therefore, no clear-cut and correct answer was given. This may also contribute to the reason why we could not detect a (stronger) effect. Likewise, we did not have access to the reasoning behind immigrants’ hypothetical healthcare-seeking behaviour. To ease the data collection, the questionnaire and the appurtenant answer categories were designed simplistically. This implied that participants could only choose one answer category instead of, for example, ranking different answer possibilities that would have provided a more nuanced understanding of the healthcare-seeking process. Further, we did not measure the participants’ understanding of the written or course materials which may a consideration for future studies. Other limitations include that we only had two settings, a control and an intervention setting. To reduce setting effects, we could have switched the two settings around in the second year of the data collection, but it was not possible, due to a lack of resources. Another limitation is the large number of participants who were lost to follow up. Additionally, we only measured the effect of the intervention on healthcare utilization in the following year, which may be inadequate, especially in a group that may have lower use initially (among others due to healthy immigrant effect). Yet, several other studies have detected changes in healthcare-seeking behavior in 1 year (or even less) [36]. Our study did not include measures of general health status of the participants, and whether their health status changed during the data collection period. This information could have helped to explain the observed actual health care use. Finally, access to out-of-hours emergency medical assistance in the Capital Region changed in January 2012 by introducing the “1813 acute telephone” where citizens have to seek medical assessment before getting permission to see an out-of-hours doctor or go to the ER. These health system changes might have led to further confusion among the participants in the study. Whether this reduced or strengthened the effect of the intervention or whether it played no role is unknown.

### In relation to international literature

Our findings underscore immigrants’ suboptimal healthcare-seeking behaviour mainly in the case of less severe symptoms (flu-like symptoms), but also more severe cases (chest pain and major depression). This may be rooted in, among other factors, lack of knowledge of accessing the healthcare system, different interpretation of symptoms or different health beliefs. Lack of knowledge was already found among the same sample [4] and among other newly arrived immigrants in Denmark [10], defined as who have stayed less than five years in host country [41], but it has also been shown in other European countries, among both newly arrived [32] and long-term stay immigrants [42]. Regarding interpretation of symptoms, previous research in the UK has shown that ethnic minorities were at least as likely to report immediate healthcare seeking in response to serious clinical vignettes (chest pain and lumps) as the white British respondents, also after controlling for interpretation of the vignette, access to health services and attitudes to health and health care [43]. However, to our knowledge, evidence is lacking as to whether variance in the interpretation of harmless/normal symptoms between immigrants and native-born Europeans exists. We did not include an ethnic Danish control or intervention group. However, many ethnic Danes also do not show optimal and appropriate healthcare-seeking behaviour or use of healthcare services [44]. Addressing health literacy and healthcare system education may be of relevance to a broader part of the population.

Assessing healthcare utilisation by counted use of services does not give us any information on whether the use was “appropriate” or “inappropriate”. There is no gold standard for number of healthcare consultations, yet, use of healthcare according to need is considered an equitable distribution of healthcare resources [45]. An English study investigating general patients’ views on “appropriate” use of services and their help seeking found that patients generally describe clear rationales for help seeking, even for seemingly minor symptoms, and that the anxiety level of a health symptom strongly predicted their healthcare-seeking behaviour [46]. Anxiety levels could be taken into consideration to understand the mechanisms behind the interpretation of symptoms among individuals and to target preventive efforts to avoid unnecessary emergency healthcare use.

Our study confirmed previous findings that written information alone has little effect [34]. In most studies, written information has proven most effective when it is supplemented with interactions between patients and professionals [47], in the same way that our study has partly shown. Researchers have stated that interventions implemented in already existing structures are likely to be more successful and sustainable [48]. Using existing settings such as language schools seems pertinent for providing healthcare education to newly arrived immigrants [33], but relevance of other settings should be further explored. It has been argued that a culturally sensitive approach is essential for the practice of immigrant health education and that health education targeting immigrant groups should be based on a thorough understanding of cultural factors [35, 49]. This is supported by a Swedish study which demonstrated that a relatively short and culturally tailored lifestyle intervention course strengthened immigrant women’s self-perceived health [32]. Owing to the well-educated and experienced professional language school teachers who are trained to teach a culturally and socioeconomically diverse group of immigrants, the healthcare education was likely to be communicated successfully to the target group even though the healthcare education was not tailored to a specific cultural group. In line with the recommendations for effective delivery of health education programmes [49], the developed material also matched the general literacy and health literacy level of the immigrants. Teachers’ motivation also has an impact on learning outcomes [50]. Some teachers found that the intervention detracted from the actual learning goals and were somewhat opposed to the intervention [51]. If healthcare education with specific learning goals becomes a compulsory part of the language school programme, a stronger effect of the healthcare education could be expected through the motivation of teachers and thereby the broader prioritisation in the organisation.

### Implications for research, policy and practice

Equal access to healthcare can only exist if immigrants are provided with information on the healthcare system [52] at the same level as the majority populations, and an appropriate use of healthcare services can serve as an indicator of the integration of immigrants within the healthcare sector. Rather than claiming that a nationwide implementation of the educational course would lead to more appropriate healthcare-seeking behaviour and use among newly arrived immigrants, we argue that implementation of the educational course as part of the language school curriculum and thereby providing information on the healthcare system is more favourable than no information. This is also supported by evaluation among the immigrants themselves reporting that information about the Danish healthcare system as part of the language school programme was important, relevant and needed [33]. Findings from this study suggest that language schools serve as a suitable setting for teaching newly arrived immigrants about the healthcare system to support their access to the healthcare system. If immigrants further correct their medical visits to the appropriate places instead of using costly emergency care, major resources are likely to be saved in the healthcare system. Yet, only providing teaching will not solve the access and utilization problem. Multi-dimensional and multi-sectional efforts that include other means as well (such as cultural mediators, development of diversity competence in healthcare, reduction of financial barriers, e.g. dental care, etc.) are needed together with a multipronged strategy to address various groups of immigrants, also long-term stay immigrants.

This study should be used as a stepping-stone towards testing different designs of improving more appropriate healthcare-seeking behaviour and utilisation of services. Lessons learnt from the process evaluation underscored the importance of participants’ existing health beliefs which challenged the underlying assumption of the intervention that increased knowledge on the structure of and access to the Danish healthcare system would lead to changed healthcare-seeking behaviour and healthcare use [3]. In support of this, a recent Australian PhD study elucidated that immigrants have a range of unrecognised health literacy skills that are not adequately understood or addressed within the healthcare system [35]. Thereby, future healthcare educational interventions should be more tailored to immigrants’ health beliefs and health needs using a strengths focus.

## Conclusions

The ability to seek healthcare when needed on an equal footing with other residents is essential. Successful integration is likely to have positive consequences for access to the healthcare system and thus health outcomes in the long term and vice versa. In the interest of not only individual immigrants and their welfare and well-being, but also the future social and economic development of society, there are good reasons to incorporate healthcare system information into integration efforts right from the beginning of immigrants’ arrival, for example, embedding them in language school programmes. This may equip immigrants to access healthcare services in an equitable manner. However, the results of this study do not give full guidance as to how we should do this given that the results do not show an effect on actual healthcare use. The results thereby underscore the need for refining and developing educational interventions and ensuring adequate utilisation of healthcare services by other means as well, including multi-dimensional and multi-sectional efforts.

## Abbreviations

ER: Emergency room
GPs: General practitioners
CPR number: Civil personal registration number
RR: Rate ratio
